# Dissection of a QTL Hotspot on Mouse Distal Chromosome 1 that Modulates Neurobehavioral Phenotypes and Gene Expression

**DOI:** 10.1371/journal.pgen.1000260

**Published:** 2008-11-14

**Authors:** Khyobeni Mozhui, Daniel C. Ciobanu, Thomas Schikorski, Xusheng Wang, Lu Lu, Robert W. Williams

**Affiliations:** Department of Anatomy and Neurobiology, University of Tennessee Health Science Center, Memphis, Tennessee, United States of America; The Wellcome Trust Centre for Human Genetics, University of Oxford, United Kingdom

## Abstract

A remarkably diverse set of traits maps to a region on mouse distal chromosome 1 (Chr 1) that corresponds to human Chr 1q21–q23. This region is highly enriched in quantitative trait loci (QTLs) that control neural and behavioral phenotypes, including motor behavior, escape latency, emotionality, seizure susceptibility (*Szs1*), and responses to ethanol, caffeine, pentobarbital, and haloperidol. This region also controls the expression of a remarkably large number of genes, including genes that are associated with some of the classical traits that map to distal Chr 1 (e.g., seizure susceptibility). Here, we ask whether this QTL-rich region on Chr 1 (*Qrr1*) consists of a single master locus or a mixture of linked, but functionally unrelated, QTLs. To answer this question and to evaluate candidate genes, we generated and analyzed several gene expression, haplotype, and sequence datasets. We exploited six complementary mouse crosses, and combed through 18 expression datasets to determine class membership of genes modulated by *Qrr1*. *Qrr1* can be broadly divided into a proximal part (*Qrr1p*) and a distal part (*Qrr1d*), each associated with the expression of distinct subsets of genes. *Qrr1d* controls RNA metabolism and protein synthesis, including the expression of ∼20 aminoacyl-tRNA synthetases. *Qrr1d* contains a tRNA cluster, and this is a functionally pertinent candidate for the tRNA synthetases. *Rgs7* and *Fmn2* are other strong candidates in *Qrr1d*. FMN2 protein has pronounced expression in neurons, including in the dendrites, and deletion of *Fmn2* had a strong effect on the expression of few genes modulated by *Qrr1d*. Our analysis revealed a highly complex gene expression regulatory interval in *Qrr1*, composed of multiple loci modulating the expression of functionally cognate sets of genes.

## Author Summary

A major goal of genetics is to understand how variation in DNA sequence gives rise to differences among individuals that influence traits such as disease risk. This is challenging. Most traits are the result of a complex interplay of genetic and environmental factors. One of the first steps in the path from DNA to these complex traits is the production of mRNA molecules. Understanding how sequence differences modulate expression of different RNAs is fundamental to understanding the molecular origins of complex traits. Here, we combine classic gene mapping methods with microarray technology to characterize and quantify RNA levels in different crosses of mice. We focused on a hotspot on chromosome 1 that controls the expression of a large number of different types of RNAs in the brain. This hotspot also controls many disease traits, including anxiety levels, and vulnerability to seizure in mice and humans. We show that this hotspot is made up of several distinct functional regions, one of which has an unusually strong and selective effect on aminoacyl-tRNA synthetases and other genes involved in protein translation.

## Introduction

The distal part of mouse Chr 1 harbors a large number of QTLs that generate differences in behavior. Open field activity [1], fear conditioning [2], rearing behavior [3], and several other measures of emotionality [4],[5] have been repeatedly mapped to distal Chr 1. This region is also notable because it appears to influence responses to a wide range of drugs including ethanol [6], caffeine [7], pentobarbital [8], and haloperidol [9]. In addition to the behavioral traits, a number of metabolic, physiological and immunological phenotypes have been mapped to this region (table 1) [10]–[36]. This QTL rich region on mouse distal Chr 1 exhibits reasonably compelling functional and genetic concordance with the orthologous region on human Chr 1q21–q23. Prime examples of genes in this region that have been associated with similar traits in mouse and human are *Rgs2* (anxiety in both species), *Apoa2* (atherosclerosis), and *Kcnj10* (seizure susceptibility) [37]–[42].

**Table 1:** Classical QTLs on Chr 1 from 172–178 Mb; listed by approximate position from proximal to distal end (adapted from Mouse Genome Informatics).

MGI ID	Symbol	Name	Type	Cross	Reference
2389129	*Bmd5*	Bone mineral density 5	bone	C3H/HeJ×C57BL/6J	[10]
1349434	*Bmd1*	Bone mineral density 1	bone	C57BL/6J×CAST/Ei	[11]
3624655	*Scgq1*	Spontaneous crescentic glomerulonephritis QTL 1	kidney	C57BL/6J×SCG/Kj	[12]
2680094	*Rrodp1*	Rotarod performance 1	behavior	129S6/SvEvTac×C57BL/6J	[13]
1891474	*Tir3c*	Trypansomiasis infection response 3c	immune	A/JOlaHsd; BALB/cJOlaHsd; C57BL/6JOlaHsd	[14]
2387316	*Elnt*	Escape latencies during navigation task	behavior	C57BL/6J×DBA/2J	[15]
1350920	*Emo1*	Emotionality 1	behavior	BALB/cJ×C57BL/6J	[5]
3050452	*Alcdp1*	Alcohol dependency 1	behavior	C57BL/6J×DBA/2J	[16]
1309452	*Alcw1*	Alcohol withdrawal 1	behavior	C57BL/6J×DBA/2J	[6]
2150827	*Cafq1*	Caffeine metabolism QTL 1	metabolism	C3H/HeJ×APN	[7]
1098770	*Szs1*	Seizure susceptibility 1	CNS	C57BL/6×DBA/2	[17]
2661242	*Cd8mts1*	CD8 T memory cell subset 1	immune	BALB/c×C3H×C57BL/6×DBA/2	[18]
3613641	*Chlq1*	Circulating hormone level QTL 1	endocrine	BALB/cJ×C3H/HeJ×C57BL/6J×DBA/2J	[19]
1345638	*Pbw1*	Pentobarbital withdrawal QTL 1	behavior	C57BL/6J×DBA/2J	[8]
2661145	*Ssta2*	Susceptibility to Salmonella typhimurium antigens 2	immune	HIII×LIII	[20]
3522039	*Trglyd*	Triglycerides	metabolism	C57BL/6J×RR	[21]
1346066	*Gvhd1*	Graft-versus-host disease 1	Immune	B10.D2-H2d×C57BL/10J	[22]
2155287	*Radpf2*	Radiation pulmonary fibrosis 2	Immune	C3H/Kam×C57BL/6J	[23]
2151854	*Pbwm*	Pentobarbital withdrawal modifier	behavior	C57BL/6J×DBA/2J	[24]
1890350	*Ath9*	Atherosclerosis 9	metabolism	C57BL/6J×FVB/NCr	[25]
2682357	*Bslm4*	Basal locomotor activity 4	behavior	BALB/cJ×C57BL/6J; C57BL/6J×DBA/2J; C57BL/6J×LP/J	[26]
1891174	*Cbm1*	Cerebellum weight 1	CNS	C57BL/6J×DBA/2J	[27]
2137602	*Cq2*	Cholesterol QTL 2	metabolism	C57BL/6J×KK-Ay	[28]
2680927	*Eila1*	Ethanol induced locomotor activity	behavior	C3H/HeJ×C57BL/6J	[29]
2660561	*Fglu2*	Fasting glucose 2	metabolism	C57BL/6J×KK-Ay	[30]
2137474	*Hpic2*	Haloperidol induced catalepsy 2	behavior	C57BL/6J×DBA/2J	[9]
1890554	*Melm2*	Melanoma modifier 2	tumor	BALB/cJ×C57BL/6J	[31]
2684308	*Mnotch*	Modifier of Notch		129X1/SvJ×C57BL/6J	[32]
2149094	*Sle9*	Systematic lupus erythematosus susceptibility 9	immune	BXSB/J×C57BL/10Ola	[33]
3579342	*Sphsr1*	Spermatocyte heat stress resistance 1	other	C57BL/6CrSlc×MRL/MpJSlc	[34]
2148991	*Yaa4*	Y-linked autoimmune acceleration	immune	BXSB/J×C57BL/10Ola	[35]
3613551	*Bglu3*	Blood glucose level 3	metabolism	C3H/HeJ×C57BL/6J	[36]

Studies of gene expression in the central nervous system (CNS) of mice have revealed major strain differences in the expression level of numerous genes located on distal Chr 1, e.g., *Copa*, *Atp1a2*, and *Kcnj9* [26], [43]–[45]. These differentially expressed genes are strong candidates for the behavioral and neuropharmacological traits that map to this region. We have recently shown that sequence variants near each of these candidate genes are often responsible for the prominent differences in expression [26],[46],[47]. In other words, sequence differences near genes such as *Kcnj9* cause expression to differ, and variation in transcript level maps back to the location of the source gene itself. Transcripts of this type are associated with *cis*-QTLs.

These expression genetic studies have also uncovered another unusual characteristic of mouse distal Chr 1. In addition to the extensive *cis*-effects, a large number of transcripts of genes located on other chromosomes map into this same short interval on distal Chr 1 [47],[48]. These types of QTLs are often referred to as *trans*-QTLs. The clustering of *trans*-QTLs to distal Chr 1 has been replicated in multiple crosses and CNS microarray datasets [47]. We refer to this region of Chr 1, extending from *Fcgr3* (172.5 Mb) to *Rgs7* (177.5 Mb) as the QTL-rich region on Chr 1, or *Qrr1*. It is possible that these modulatory effects on expression are the first steps in a cascade of events that are ultimately responsible for many of the prominent differences in behavior and neuropharmacology. For example, *Qrr1* modulates the expression of several genes that have been implicated in seizure (e.g., *Scn1b*, *Pnpo*, *Cacna1g*), and this may be a basis for the strong influence *Qrr1* has on seizure susceptibility [41].

In this study, we exploited 18 diverse array datasets derived from different mouse crosses to systematically dissect the expression QTLs in *Qrr1*. The strong *trans* effects are consistently detected in CNS tissues of C57BL/6J (B6)×DBA/2J (D2) and B6×C3H/HeJ (C3H) crosses, but are largely absent in ILS/Ibg (ILS)×ISS/Ibg (ISS) and C57BL/6By (B6y)×BALB/cBy (BALB), and in all non-neural tissues we have examined. We applied high-resolution mapping and haplotype analysis of *Qrr1* using a large panel of BXD recombinant inbred (RI) strains that included highly recombinant advanced intercross RI lines. Our analyses revealed multiple distinct loci in *Qrr1* that regulate gene expression specifically in the CNS. The distal part of *Qrr1* (*Qrr1d*) has a strong effect on the expression of numerous genes involved in RNA metabolism and protein synthesis, including more than half of all aminoacyl-tRNA synthetases. *Fmn2* and *Rgs7*, and a cluster of tRNAs are the strongest candidates in *Qrr1d*.

## Results

### Enrichment in Classical QTLs

The Chr 1 interval, from 172–178 Mb, harbors 32 relatively precisely mapped QTLs for classical traits such as alcohol dependency, escape latency, and emotionality (Mouse Genome Informatics at www.informatics.jax.org, Table 1). To compare the enrichment of QTLs in *Qrr1* with that in other regions, we counted classical QTLs in 100 non-overlapping intervals covering almost the entire autosomal genome (table S1). These intervals were selected to contain the same number of genes as *Qrr1*. Numbers of QTLs ranged from 0 to 23, and averaged at 9.16±5.37 (SD). Compared to these regions, *Qrr1* had the highest QTL number, over 4 SD above the mean, and over three times higher than average.

### Enrichment in Expression QTLs in Neural Tissues

In this section, we summarize the number of expression phenotypes that map to *Qrr1* in different tissues and mouse crosses. The results are based on the analysis of 18 array datasets that provide estimates of global mRNA abundance in neural and non-neural tissues from six different crosses. These crosses are—(i) BXD RI and advanced intercross RI strains derived from B6 and D2, (ii) CXB RI strains derived from B6y×BALB, (iii) LXS RI strains derived from ILS and ISS, (iv) B6×C3H F2 intercrosses, and (v & vi) two separate B6×D2 F2 intercrosses. These datasets were generated by collaborative efforts over the last few years [46], [47], [49]–[52] and some were generated more recently (e.g., the Illumina datasets for BXD striatum and LXS hippocampus, and BXD Hippocampus UMUTAffy Exon Array dataset). All datasets can be accessed from GeneNetwork (www.genenetwork.org).

We mapped loci that modulate transcript levels and selected only those transcripts that have peak QTLs in *Qrr1* with a minimum LOD score of 3. This corresponds to a generally lenient threshold with genome-wide *p*-value of 0.1 to 0.05, but corresponds to a highly significant pointwise *p*-value. Because we are mainly interested in testing a short segment on Chr 1, a pointwise (region-wise) threshold is more appropriate to select those transcripts that are likely to be modulated by *Qrr1*. *Qrr1* covers approximately 0.2% of the genome and extends from *Fcgr3* (more precisely, SNP rs8242852 at 172.887364 Mb using Mouse Genome Assembly NCBI m36, UCSC Genome Browser mm8) through to *Rgs7* (SNP rs4136041 at 177.273526 Mb). We defined this region on the basis of the large number of transcripts that have maximal LOD scores associated with markers between these SNPs.

Hundreds of transcripts map to *Qrr1* with LOD scores ≥3 in neural tissue datasets of BXD RI strains, B6D2F2 intercrosses, and B6C3HF2 intercrosses (table 2). The QTL counts in *Qrr1* are far higher than the average of 15 to 35 expression QTLs in a typical 6 Mb interval. The fraction of QTLs in *Qrr1* is as high as 14% of all *trans*-QTLs, and 5% of all *cis*-QTLs in the whole genome (table 2). The enrichment in *trans*-QTLs in *Qrr1* is even more pronounced when the QTL selection stringency is increased to a LOD threshold of 4 (genome-wide *p*-value of approximately 0.01). For example, 27% of all highly significant *trans*-QTLs in the BXD cerebellum dataset are in *Qrr1* (table 2). The BXD hippocampus dataset that was assayed on the Affymetrix Exon ST array is an exception—there are over a million probe sets in this array, and the percent enrichment of QTLs in *Qrr1* appears to be relatively low. Nevertheless, about 1000 transcripts map to *Qrr1* in this exon dataset.

**Table 2:** Expression QTLs in *Qrr1* in different crosses and tissues.

Cross	N^a^	Dataset & Normalization	Tissue	Array	LOD≥3				LOD≥4	
					trans^b^	cis^b^	% trans^c^	% cis^d^	% trans^c^	% cis^d^
B6D2F2	58	OHSU/VA (Sep05) PDNN	Striatum	Affymetrix M430v2	197	56	8	5	18	5
B6D2F2	56	OHSU/VA mRNA (Aug05) PDNN	Whole brain	Affymetrix M430	79	30	1	2	5	2
BXD	45	SJUT (Mar05) PDNN	Cerebellum	Affymetrix M430	439	44	9	2	27	2
BXD	69	Hippocampus Consortium (Dec05) PDNN	Hippocampus	Affymetrix M430v2	345	54	7	1	22	1
BXD	39	INIA (Jan06) PDNN	Forebrain	Affymetrix M430	279	39	5	1	13	1
BXD	64	Hamilton Eye Institute (Sep06) RMA	Eye	Affymetrix M430v2	156	43	2	1	2	1
BXD	54	HQF (Nov 07) RankInv	Striatum	Illumina M6.1	97	31	1	1	2	1
BXD	29	HBP/Rosen(Apr05) PDNN	Striatum	Affymetrix M430v2	94	25	2	1	6	1
BXD	63	UMUTAffy RMA (Mar08)	Hippocampus	Affymetrix Exon 1.0 ST	700	302	0.4	1	0.5	1
BXD	40	UNC (Jan06) BothSexes LOWESS	Liver	Agilent G4121A	9	20	0.3	1	0.7	1
BXD	53	Kidney Consortium (Aug06) PDNN	Kidney	Affymetrix M430v2	8	33	0.2	1	0	1
BXD	30	GNF (Mar03) MAS5	Hematopoietic Cells	Affymetrix U74Av2	0	6	0	3	0	3
LXS	75	NIAAA INIA (May07) RankInv	Hippocampus	Illumina M6.1	10	28	0.4	1	1	1
B6C3F2	238	UCLA BHHBF2 (2005) mlratio	Brain	Agilent	516	51	14	3	23	2
B6C3F2	306	UCLA BHHBF2 (2005) mlratio	Muscle	Agilent	15	33	0.3	2	0.3	2
B6C3F2	298	UCLA BHHBF2 (2005) mlratio	Liver	Agilent	63	46	0.7	3	0.6	3
B6C3F2	282	UCLA BHHBF2 (2005) mlratio	Adipose	Agilent	56	34	0.5	3	0.4	3
CXB	13	Hippocampus Consortium (Dec05) PDNN	Hippocampus	Affymetrix M430v2	7	12	0.08	2	0.1	2

a Number of RI strains or F2 mice.

b Number of *cis*- and *trans*-QTLs in *Qrr1* at minimum LOD of 3; complete list of these transcripts can be retrieved from www.genenetwork .org using search key “LRS = (15 500 Chr1 172 178)”.

c Percent of *trans*-QTLs in *Qrr1* = [(number of *trans*-QTLs in *Qrr1*)/(total number of *trans*-QTLs in the whole genome)×100].

d Percent of *cis*-QTLs in *Qrr1* = [(number of *cis*-QTLs in *Qrr1*)/(total number of *cis*-QTLs in the whole genome)×100].

In contrast to the CNS datasets, relatively few transcripts map to *Qrr1* in non-neural tissues of the BXD strains and B6C3HF2 intercrosses. While the number of *cis*-QTLs is still relatively high (1–3%), *Qrr1* has limited or no *trans*-effect in these datasets (table 2).

*Qrr1* does not have a strong *trans*-effect in the LXS and CXB hippocampus datasets (table 2). This indicates that the sequence variants underlying the *trans*-QTLs do not segregate to nearly the same extent in the LXS and CXB RI panels as they do in B6×D2 and B6×C3H crosses. This contrast among crosses can be exploited to parse *Qrr1* into sub-regions and identify stronger candidate genes.

### Replication of *trans*-QTLs in Multiple Datasets

**Figure 1:**
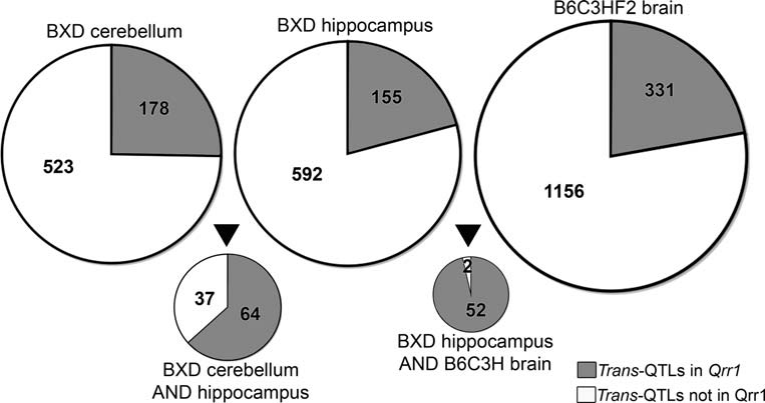
Highly replicable trans-QTLs in *Qrr1*. The charts illustrate the total number of *trans*-QTLs (LOD≥4) in *Qrr1* (shaded) and in other regions of the genome (non-shaded) in three datasets—BXD cerebellum, BXD hippocampus, and B6C3H F2 brain. The smaller charts represent the *trans*-QTLs in BXD hippocampus that are also detected in BXD cerebellum, and B6C3HF2 brain datasets. Out of the 101 *trans*-QTLs common to both BXD hippocampus and cerebellum, 64 are in *Qrr1* and the remaining 37 are located in other regions of the genome. The BXD hippocampus and B6C3HF2 brain datasets have 54 common *trans*-QTLs, and almost all (52 out of 54) are in *Qrr1*.

The *trans*-QTLs in *Qrr1* are highly replicable. A large fraction of the transcripts, in some cases represented by multiple probes or probe sets, map to *Qrr1* in multiple CNS datasets. For example, there are 747 unique *trans*-QTLs with LOD scores greater than 4 (genome-wide *p*-value≤0.01) in the BXD hippocampus dataset (assayed on Affymetrix M430v2 arrays). Out of these highly significant *trans*-QTLs, 155 are in *Qrr1* and the remaining 592 are distributed across the rest of the genome (figure 1). We compared the *trans*-QTLs in the hippocampus dataset with a similar collection of *trans*-QTLs (LOD≥4) in the cerebellum dataset (assayed on Affymetrix M430 arrays). Only 101 *trans*-QTLs in the hippocampus are replicated in the cerebellum (for *trans*-QTLs that were declared as common, the average distance between peak QTL markers in the two datasets is 1.6 Mb). But it is remarkable that of the subset of common *trans*-QTLs, 64 are in *Qrr1* (figure 1). The replication rate of *trans*-QTLs in *Qrr1* is therefore about 6-fold higher relative to the rest of the genome. When we compared the BXD hippocampus dataset with the B6C3HF2 brain dataset (assayed on Agilent arrays), we found 54 *trans*-QTLs common to both datasets (for the common *trans*-QTLs, the average distance between peak markers in the two datasets is 2.7 Mb). Strikingly, out of the 54 *trans*-QTLs common to both crosses, 52 are in *Qrr1* (figure 1).

Among the transcripts with the most consistent *trans*-QTLs are glycyl-tRNA synthetase (*Gars*), cysteinyl-tRNA synthetase (*Cars*), asparaginyl-tRNA synthetase (*Nars*), isoleucyl tRNA synthetase (*Iars*), asparagine synthetase (*Asns*), and activating transcription factor 4 (*Atf4*). These transcripts map to *Qrr1* in almost all datasets in which the strong *trans*-effect is detected. *Gars*, *Cars*, and *Nars* are aminoacyl-tRNA synthetases (ARS) that charge tRNAs with amino acids during translation. *Asns* and *Atf4* are also involved in amino acid metabolism—*Asns* is required for asparagine synthesis and is under the regulation of *Atf4*, which in turn is sensitive to cellular amino acid levels [53]. Other transcripts that consistently map as *trans*-QTLs to *Qrr1* include brain expressed X-linked 2 (*Bex2*), splicing factor *Sfrs3*, ribonucleoproteins *Snrpc* and *Snrpd1*, ring finger protein 6 (*Rnf6*), and RAS oncogene family member *Rab2*.

### Candidates in *Qrr1*

*Qrr1* contains 164 known genes. The proximal part of *Qrr1* is gene-rich and has several genes with high expression in the CNS (e.g. *Pea15*, *Kcnj9*, *Kcnj10*, *Atp1a2*). The middle to distal part of *Qrr1* is relatively gene sparse and consists mostly of clusters of olfactory receptors and members of the interferon activated *Ifi200* gene family. Though comparatively gene sparse, the middle to distal part of *Qrr1* contains a small number of genes that have high expression in the CNS—*Igsf4b*, *Dfy*, *Fmn2*, and *Rgs7*.

A subset of 35 genes were initially selected as high priority candidates based on the number of known and inferred sequence differences between the B6 allele (*B*) and D2 allele (*D*) and based on expression levels in multiple CNS datasets (table 3). Eleven of these candidates contain missense SNPs segregating in B6×D2 crosses. We also scanned *Qrr1* for variation in copy number [54],[55]. Graubert et al. [55] reported segmental duplication in *Qrr1* with a copy number gain in D2 compared to B6 near the intelectin 1 (*Itlna*) gene at 173.352 Mb. We failed to detect any expression signatures of a copy number variation around *Itlna* in any of the GeneNetwork datasets. However, we did identify an apparent 150 kb deletion across the *Ifi200* gene cluster (175.584–175.733 Mb). Affymetrix probe sets 1426906_at, 1452231_x_at, and 1452349_x_at detect *Ifi204* and *Mnda* transcripts in B6 but not in D2. The expression difference is robust enough to generate *cis*-QTLs with very high LOD scores (>40). This gene cluster has low expression in the CNS (Affymetrix declares this probe sets to be “not present”), but high expression in tissues such as hematopoietic stem cells and kidney, in which the *trans*-effect of *Qrr1* is not detected. The *Ifi200* gene cluster was therefore excluded as a high priority candidate.

**Table 3:** Candidate genes in *Qrr1*.

Gene	Mb	nsSNP^a^	Exp^b^	BXD^c^	B6C3HF2^c^	CXB^c^	LXS^c^
*Fcgr3*	172.981	2	8.2				*cis*
*Sdhc*	173.059	2	12.3	*cis*			*cis*
*Pcp4l1*	173.103		8.7	*cis*	*cis*		
*Tomm40l*	173.148		9.67	*cis*		*cis*	
*Apoa2*	173.155		7.2		*cis*	*cis*	*cis*
*Fcer1g*	173.160		8.5	*cis*			*cis*
*Ndufs2*	173.165	2	13.6	*cis*			
*Adamts4*	173.181	1	8.1	*cis*	*cis*	*cis*	*cis*
*B4galt3*	173.201		9.5	*cis*			
*Ppox*	173.207		7.8	*cis*	*cis*		*cis*
*Usp21*	173.212		9.0				*cis*
*Ufc1*	173.219		10.8	*cis*	*cis*	*cis*	*cis*
*Dedd*	173.260		9.7	*cis*			
*Nit1*	173.272	1	9.8	*cis*	*cis*	*cis*	*cis*
*Pfdn2*	173.276		12.8	*cis*	*cis*	*cis*	
*Arhgap30*	173.319	4	7.6				
*Usf1*	173.342		7.5	*cis*	*cis*		*cis*
*Refbp2*	173.434	2	9.7		*cis*		*cis*
*Vangl2*	173.935		7.6	*cis*	*cis*	*cis*	*cis*
*Ncstn*	173.996		8.5		*cis*		*cis*
*Copa*	174.013	1	12.7	*cis*	*cis*		*cis*
*Pex19*	174.057	1	9.9	*cis*		*cis*	*cis*
*Wdr42a*	174.078		10.3	*cis*	*cis*		
*Pea15*	174.127		14.1		*cis*		
*Atp1a2*	174.202		15.4	*cis*	*cis*	*cis*	*cis*
*Igsf8*	174.243		12.1	*cis*			
*Kcnj9*	174.251		9.1	*cis*	*cis*	*cis*	*cis*
*Kcnj10*	174.271	1	11.2	*cis*	*cis*	*cis*	
*Tagln2*	174.430		8.8				
*Dusp23*	174.561		7.4		*cis*		
*Dfy*	175.262		10.3	*cis*		*cis*	*cis*
*Igsf4b*	175.264		10.6	*cis*			
*Fmn2*	176.419	3	10.4	*cis*	*cis*	*cis*	
*Grem2*	176.764		8.2	*cis*			
*Rgs7*	176.989		11.5	*cis*	*cis*		

a Number of missense mutations between *B* and *D* alleles.

b Mean expression signal of probe sets in BXD Hippocampus PDNN dataset; below 7 is considered to be below background.

c *Cis*-QTLs in BXD, B6C3HF2, CXB, and LXS crosses.

### *cis*-QTLs in *Qrr1*

Transcripts of 26 of the 35 selected candidate genes map as *cis*-QTLs (LOD≥3) in the BXD CNS datasets (table 3). These putatively *cis*-regulated genes are among the strongest candidates in the QTL interval. The *D* allele in *Qrr1* has the positive effect on the expression of *Sdhc*, *Ndufs2*, *Adamts4*, *Dedd*, *Pfdn2*, *Ltap*, *Pea15*, *Atp1a2*, *Kcnj9*, *Kcnj10*, *Igsf4b*, and *Grem2*. Increase in expression caused by the *D* allele ranges from about 10% for *Adamts4* to over 2-fold for *Atp1a2*. In contrast, the *B* allele has the positive effect on the expression of *Pcp4l1*, *Fcer1g*, *B4galt3*, *Ppox*, *Ufc1*, *Nit1*, *Usf1*, *Copa*, *Pex19*, *Wdr42a*, *Igsf8*, *Dfy*, *Fmn2*, and *Rgs7*. Increase in expression caused by the *B* allele ranges from about 7% for *Usf1* to 40% for *Pex19*.

Individual probes were screened to assess if the strong *cis*-effects are due to hybridization artifacts caused by SNPs in probe targets. Thirteen candidate genes with *cis*-QTLs were then selected for further analysis and validation of *cis*-regulation by measuring allele specific expression (ASE) difference [56]. This method exploits transcribed SNPs, and uses single base extension to assess expression difference in F1 hybrids. By means of ASE, we validated the *cis*-regulation of 10 candidate genes—*Ndufs2*, *Nit1*, *Pfdn2*, *Usf1*, *Copa*, *Atp1a2*, *Kcnj9*, *Kcnj10*, *Dfy*, and *Fmn2* (table 4). *Adamts4* and *Igsf4b* failed to show significant allelic expression difference. In the case of *Ufc1*, the polarity of the allele effect failed to agree with the ASE result (*D* positive at *p*-value = 0.02).

**Table 4:** Validation of *cis*-QTLs by measuring allele specific expression difference.

Gene	ProbeSet ID	SNP ID	Cis-LOD	Add. effect (QTL)^a^	High allele (ASE)	P-value
*Ndufs2*	1451096_at	rs8245216	12	0.172	*D*	2.4×10^−5^
*Adamts4*	1455965_at	rs31537832	25	−0.376		0.2
*Ufc1*	1416327_at	rs13470410	21	−0.262	*D*	0.02
*Nit1*	1417468_at	rs31552469	15	−0.154	*B*	0.01
*Pfdn2*	1421950_at	rs31549998	5	0.174	*D*	4.1×10^−7^
*Usf1*	1426164_a_at	rs31542370	5	−0.166	*B*	0.004
*Copa*	1415706_at	rs13461812	9	−0.148	*B*	3.9×10^−5^
*Atp1a2*	1455136_at	rs31570902	49	1.186	*D*	0.02
*Kcnj9*	1450712_at	rs31569118	19	0.511	*D*	0.01
*Kcnj10*	1419601_at	rs30789204	28	0.349	*D*	0.003
*Dfy*	1432273_a_at	rs31616337	24	−0.337	*B*	0.006
*Igsf4b*	1418921_at	rs31613626	7	0.171		0.3
*Fmn2*	1450063_at	rs33800912	17	−0.286	*B*	5.5×10^−6^

a Additive effect is computed as [(mean expression in *DD* homozygote)−(mean expression in *BB* homozygote)]/2 on a log_2_ scale. Positive value means *D* high expression, and negative value means *B* high expression.

### High-Resolution *cis*-QTL Mapping

**Figure 2:**
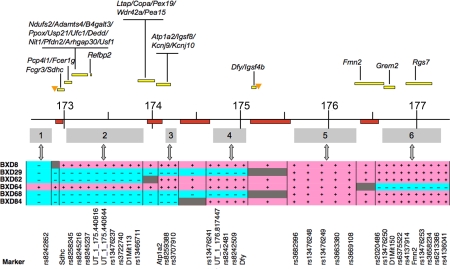
Haplotype maps of *Qrr1* recombinant BXD strains. BXD8, BXD29, BXD62, BXD64, BXD68, and BXD84 have recombinations in *Qrr1*. *B* haplotype is assigned blue (−), *D* haplotype is assigned pink (+), and recombination regions are shown in grey. The *Qrr1* interval (in Mb scale) is shown above and approximate positions of recombination are highlighted (red). The recombinant strains collectively divide *Qrr1* into six segments (labeled 1–6), and provide six sets of informative markers. Markers are shown below and approximate positions of candidate genes (yellow bars) and tRNA clusters (orange triangles) are indicated.

The BXD CNS datasets were generated from a combined panel of conventional RI strains and advanced RI strains that were derived by inbreeding advanced intercross progeny. The advanced RIs have approximately twice as many recombinations compared to standard RIs and the merged panel offers over a 3-fold increase in mapping resolution [57]. This expanded RI set combined with the relatively high intrinsic recombination rate within *Qrr1* [58] provides comparatively high mapping resolution. Mapping precision can be empirically determined by analyzing *cis*-QTLs in multiple large datasets, particularly the BXD Hippocampus Consortium, UMUTAffy Hippocampus, and Hamilton Eye datasets. These three datasets were selected because they have expression measurements from six BXD strains with recombinations in *Qrr1*. These strains—BXD8, BXD29, BXD62, BXD64, BXD68, and BXD84—collectively provide six sets of informative markers and divide *Qrr1* into six non-recombinant segments, labeled as segments 1–6 (haplotype structures shown in figure 2).

**Figure 3:**
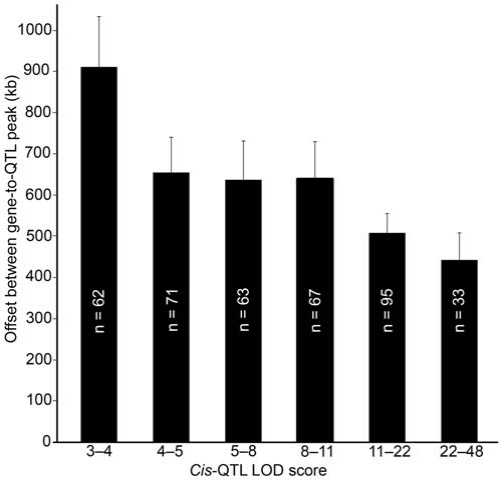
QTL mapping precision in *Qrr1*. Mapping precision was empirically determined by measuring the distance between a *cis*-QTL peak and location of parent gene. *Cis*-QTLs in BXD Hippocampus Consortium, UMUTAffy Hippocampus, and Hamilton Eye datasets were used for this purpose. Mean gene-to-QTL peak distance (y-axis) was plotted as a function of LOD score (LOD score range on x-axis). Number of probe sets in each LOD range is shown. Mapping precision increases with increase in LOD score. The mean offset for *cis*-QTLs with LOD scores 3–4 (genome-wide adjusted p-value of 0.1–0.01) is 900 kb, and the offset decreases to 650 kb at 4–5 LOD scores (p-value of 0.01–0.001). *Cis*-QTLs with LOD scores greater than 11 (p-value<10^−6^) have mean offset of only 450 kb.

As *cis*-acting regulatory elements are usually located within a few kilobases of a gene's coding sequence [59], we used the *cis*-QTLs as an internal metric of mapping precision by measuring the offset distance between a *cis*-QTL (position of peak QTL marker) and the parent gene (figure 3). For *cis*-QTLs with LOD scores between 3–4 (genome-wide *p*-value of 0.1–0.01) the mean gene-to-QTL peak distance is 900 kb. The offset decreases to a mean of 640 kb for *cis*-QTLs with LOD scores greater than 4 (*p*-value<0.001). Very strong *cis*-QTLs with LOD scores greater than 11 (*p*-value<10^−6^) have a mean gene-to-QTL peak distance of only 450 kb. In all, 60% of *cis*-QTLs we examined have peak linkage on markers located precisely in the same non-recombinant segment as the parent gene, and 30% have peak linkage on markers in a segment adjacent to the parent gene (dataset S1). These *cis*-QTLs provide an empirical metric of mapping precision within *Qrr1*.

### Parsing *trans*-QTLs by High-Resolution Mapping and Gene Functions

**Figure 4:**
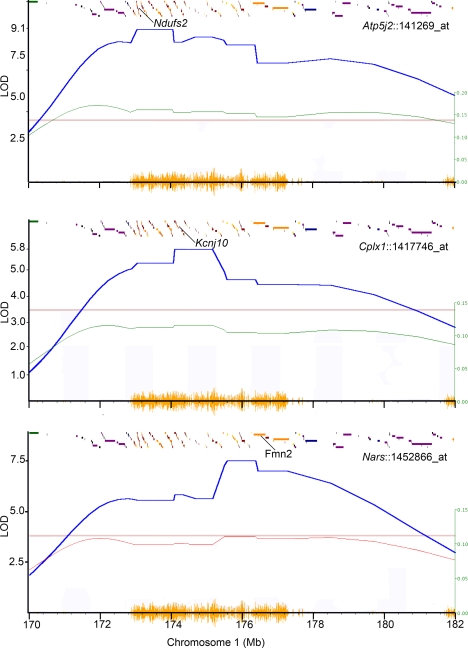
Segregation of *trans*-QTLs in *Qrr1*. Expression of *Atp5j2*, *Cplx2*, and *Nars* are modulated by *trans*-QTLs in *Qrr1* (blue plot). *D* allele has the positive additive effect (green plot; allele effect scale shown on the right) on the expression of *Atp5j2* and *Cplx2*; peak LOD scores are on markers near candidate genes *Ndufs2* and *Kcnj10*. *B* allele has the positive additive effect (red plot) on the expression of *Nars*; peak LOD score is on markers near candidate gene *Fmn2*. The horizontal lines indicate the genome-wide significant thresholds (*p*-value = 0.05). Yellow seismograph tracks the SNP density between *B* and *D* alleles. Affymetrix probe set ID for each transcript in the BXD hippocampus dataset is shown.

**Figure 5:**
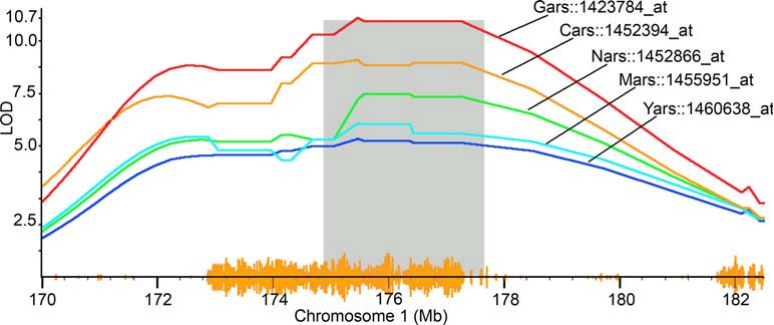
QTL for aminoacyl-tRNA synthetases in distal *Qrr1*. Transcripts of *Gars*, *Cars*, *Nars*, *Mars*, and *Yars* map as *trans*-QTLs to *Qrr1* at LOD>4 (genome-wide *p*-value<0.01) in the BXD hippocampus dataset. The *trans*-QTLs have peak LOD precisely on markers in distal part of *Qrr1*, ∼175–177.5 Mb (shaded regions). Yellow seismograph on Chr 1 (x-axis) tracks SNP density between B and D alleles. Affymetrix probe set ID for each transcript is shown.

Mapping precision of *cis*-QTLs is comparatively higher in the BXD hippocampus dataset (average offset of only 410 kb), and we used this set to examine the *trans*-QTLs (LOD≥3) at higher resolution. The *trans*-QTLs in *Qrr1* were parsed into subgroups based on the location of peak LOD score markers (figure 4). This method of resolving *trans*-QTLs effectively grouped subsets of transcripts into functionally related cohorts. For instance, all the QTLs for the aminoacyl-tRNA synthetases (ARS) have peak LOD scores only within the distal three segments of *Qrr1* (figure 5). This consistency in QTL peaks for transcripts of the same gene family is itself a good indicator of mapping precision. In addition to the ARS, numerous other genes involved in amino acid metabolism and translation map to the distal part of *Qrr1* (e.g., *Atf4*, *Asns*, *Eif4g2*, and *Pum2*).

We divided the *trans*-QTLs into two broad subgroups—those with peak QTLs on markers in the proximal part of *Qrr1* (*Qrr1p*; 172–174.5 Mb or segments 1, 2, 3 in figure 2), and those with peak QTLs on markers in the distal part of *Qrr1* (*Qrr1d*; 174.5–177.5 Mb or segments 4, 5, and 6 in figure 2). While *Qrr1p* is relatively gene-rich, only 35% of the *trans*-QTLs (129 out of 365 probe sets) have peak LOD scores in this region. The majority of *trans*-QTLs—about 65% (236 out of 365 probe sets)—have peak QTLs in the relatively gene-sparse *Qrr1d*.

The two subsets of transcripts—those with *trans*-QTLs in *Qrr1p* and those with *trans*-QTLs in *Qrr1d*—were analyzed for overrepresented gene functions using the DAVID functional annotation tool (http://david.abcc.ncifcrf.gov/). This revealed distinct gene ontology (GO) categories enriched in the two subsets (dataset S2). Enriched GOs among the transcripts modulated by *Qrr1p* include GTPase-mediate signal transduction (modified Fisher's exact test *p* = 0.001), and structural constituents of ribosomes (*p* = 0.003). Transcripts modulated by *Qrr1d* are highly enriched in genes involved in RNA metabolism (*p* = 4×10^−7^), tRNA aminoacylation (*p* = 1×10^−5^) and translation (*p* = 2×10^−5^), RNA transport (*p* = 0.003), cell cycle (*p* = 0.004), and ubiquitin mediated protein catabolism (*p* = 0.006). Other GO categories show enrichment in both *Qrr1p* and *Qrr1d*. For example, genes involved in RNA metabolism and ubiquitin-mediated protein catabolism are also overrepresented among the transcripts modulated by *Qrr1p* (*p* = 0.002 for RNA metabolism and *p* = 0.005 for ubiquitin-protein ligases). This may either be due to limitations in QTL resolution, or due to multiple loci in *Qrr1p* and *Qrr1d* controlling these subsets of transcripts.

### An Aminoacyl-tRNA Synthetase *trans*-QTL in Distal *Qrr1*

A remarkable number of transcripts of the ARS gene family map to *Qrr1*. A total of 16 ARS transcripts have *trans*-QTLs at a minimum LOD score of 3 in one or multiple BXD, B6D2F2, and B6C3H CNS datasets (table 5). In almost all cases, QTLs peak on markers on the distal part of *Qrr1*. Except for *Hars*, the *B* allele in *Qrr1* consistently increases expression by 10% to 30%. In the case of *Hars*, the *D* allele has the positive additive effect and increases expression by about 10%.

**Table 5:** Transcripts of aminoacyl tRNA synthetases that have *trans*-QTLs in *Qrr1* (LOD≥3) in one or multiple CNS datasets.

Gene	Name	ProbeID^a^	Chr^b^	Dataset^c^	LOD^d^	B/D^e^
*Nars*	asparaginyl-tRS	1452866_at_A	Chr 18	BXD cerebellum	12.0	*B*
*Gars*	glycyl-tRS	1423784_at	Chr 6	BXD hippocampus	10.6	*B*
*Rars*	arginyl-tRS	1416312_at_A	Chr 11	BXD forebrain	8.9	*B*
*Cars*	cysteinyl-tRS	10024406001	Chr 7	B6C3HF2 brain	8.9	*B*
*Yars*	tyrosyl-tRS	10024399842	Chr 4	B6C3HF2 brain	8.0	*B*
*Iars*	isoleucine-tRS	1426705_s_at	Chr 13	BXD cerebellum	7.8	*B*
*Sars*	seryl-tRS	1426257_a_at	Chr 3	BXD cerebellum	6.9	*B*
*Mars*	methionine-tRS	1455951_at	Chr 10	BXD hippocampus	6.5	*B*
*Hars*	histidyl-tRS	1438510_a_at	Chr 18	BXD hippocampus	5.2	*D*
*Iars2*	isoleucine-tRS	1426735_at	Chr 1	BXD hippocampus	4.3	*B*
*Tars*	threonyl-tRS	10024395655	Chr 15	B6C3HF2 brain	4.0	*B*
*Aars*	alanyl-tRS	1451083_s_at	Chr 8	BXD eye	3.9	*B*
*Lars*	leucyl-tRS	1448403_at_A	Chr 18	BXD cerebellum	3.7	*B*
*Ears2*	glutmyl-tRS	ILM5290446	Chr 7	BXD ILM striatum	3.7	*B*
*Aarsd1*	alanyl-tRS domain 1	1424006_at	Chr 11	B6D2F2 brain	3.5	*B*
*Dars*	aspartyl-tRS	1423800_at_A	Chr 1	BXD cerebellum	3.2	*B*

a Probe/Probe set ID.

b Physical location of gene; *Iars2* is located on Chr 1 at 186.9 Mb, and *Dars* on Chr 1 at 130 Mb.

c Dataset in which transcript has highest *trans*-QTL in *Qrr1*.

d Highest LOD score in *Qrr1*.

e Allele that increases expression.

We examined all probes or probe sets that target ARS and ARS-like genes in the B6×D2 CNS datasets. The Affymetrix platform measures the expression of 34 ARS and ARS-like genes; 24 of these map to *Qrr1* at LOD scores ranging from a low of 2 to a high of 12. Even in the case of the suggestive *trans*-QTLs (i.e., LOD values between 2 and 3), the *B* allele in *Qrr1* has the positive effect on expression. The ARS family is also highly represented among *trans*-QTLs in the B6C3HF2 brain dataset. Thirty-seven probes in this dataset target the tRNA synthetases, eleven of these have *trans*-QTLs in *Qrr1d* (LOD scores ranging from 2 to 20), and almost all have a *B* positive additive effect (exceptions are *Hars* and *Qars*). The co-localization of *trans*-QTLs to *Qrr1d*, the general consensus in parental allele effect, and their common biological function indicate that there is a single QTL in the distal part of *Qrr1* modulating the expression of the ARS. It is crucial to note that this genetic modulation is only detected in CNS tissues.

In the LXS hippocampus dataset, *Qrr1* has only a limited *trans*-effect on gene expression. Despite the weak effect, expression of *Dars2* (probe ID ILM580427) maps to the distal part of *Qrr1* at a LOD of 3. Although this is only a weak detection of the ARS QTL in the LXS dataset, it nonetheless demonstrates the strong regulatory effect of *Qrr1* on the expression of this gene family. In the case of the CXB hippocampus dataset, not a single *trans*-QTL for the ARS is detected in *Qrr1*.

### *trans*-QTLs for Transcripts Localized in Neuronal Processes

In addition to the high overrepresentation of transcripts involved in translation and RNA metabolism, several transcripts known to be transported to neuronal processes or involved in RNA transport also map to *Qrr1d*, including *Camk2a*, *Bdnf*, *Cdc42*, *Eif4e*, *Eif4g2*, *Hnrpab*, *Ppp1cc*, *Pabpc1*, *Eif5*, *Kpnb1*, *Rhoip3*, *Stau2*, and *Pum2* [60]–[63]. An interesting example is provided by the brain derived neurotrophic factor (*Bdnf*). Two alternative forms of *Bdnf* mRNA are known—one isoform has a long 3′ UTR and is specifically transported into the dendrites; the other isoform has a short 3′ UTR and remains primarily in the somatic cytosol [64]. The Affymetrix M430 arrays contain two different probe sets that target these *Bdnf* isoforms. Probe set 1422169_a_at targets the distal 3′ UTR and is essentially specific for the dendritic isoform, and probe set 1422168_a_at targets a coding sequence common to both isoforms. Although both probe sets detect high expression signal in the hippocampus, only the dendritic isoform maps as a *trans*-QTL to *Qrr1d*. This enrichment in transcripts that are transported to neuronal processes raises the possibility that this CNS specific *trans*-effect may be related to local protein synthesis.

### tRNAs in *Qrr1*

Prompted by the many ARS transcripts that consistently map to *Qrr1d*, we searched the genomic tRNA database [65] for tRNAs in this region. Interestingly, distal Chr 1 is one of many tRNA hotspots in the mouse genome and several predicted tRNAs are clustered in the non-coding regions of *Qrr1* (figure 2). The majority of these tRNA sequences are in the proximal end of *Qrr1*, over 2 Mb away from *Qrr1d*. We scanned the intergenic non-coding regions in *Qrr1d* for tRNAs using the tRNAscan-SE software [65] and uncovered tRNAs for arginine and serine, and three pseudo-tRNA sequences between genes *Igsf4b* and *Aim2* (175.204–175.257 Mb) in *Qrr1d* (dataset S3). Transfer RNAs are involved in regulating transcription of the ARS in response to cellular amino acid levels [66] and are functionally highly relevant candidates in *Qrr1d*. Polymorphism in the tRNA clusters (e.g., possible copy number variants, differences in tRNA species) may have significant impact on the expression of the ARS.

### Sequence Analysis of Crosses

*Trans*-regulation of large number of transcripts by *Qrr1* is a strong feature of crosses between B6 and D2—both the BXD RI set and B6D2F2 intercrosses—and in the B6 and C3H intercrosses. The feature is much weaker in the large LXS RI set and in the small CXB panel. The effect specificity demonstrates that a major source of the *Qrr1* signal is generated by variations between *B* and *D*, and *B* and C3H alleles (*H*) but not by variations between the ILS and ISS alleles (*L* and *S*, respectively), and *B* and BALB alleles (*C*). This contrast can be exploited to identify sub-regions that underlie the *trans*-QTLs [67].

**Figure 6:**
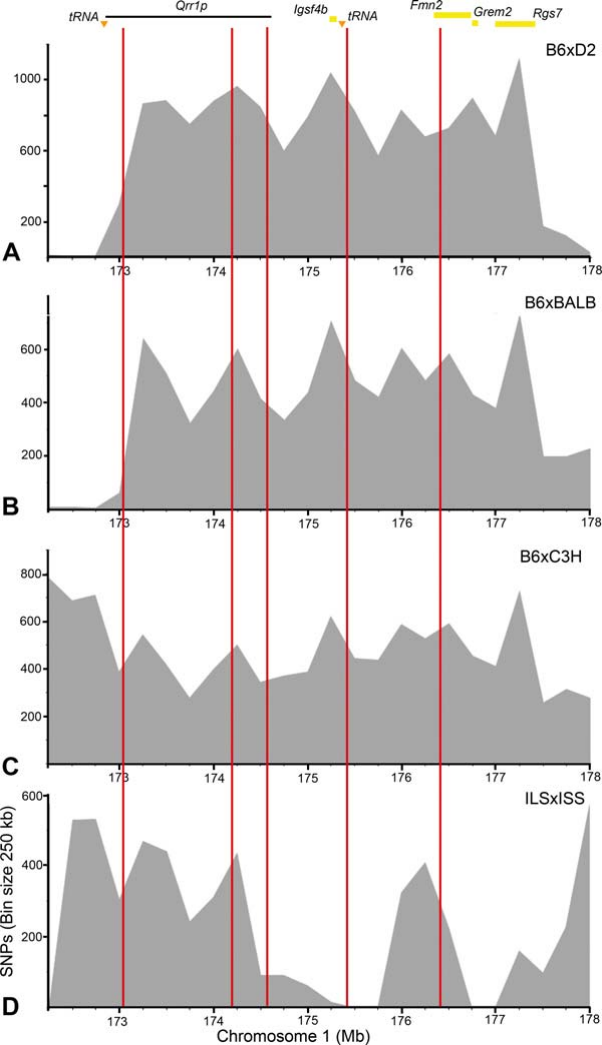
SNP comparison between crosses. SNPs in *Qrr1* were counted for (A) C57BL/6J (B6)×DBA/2J (D2), (B) B6×BALB/cBy (BALB), (C) B6×C3H/HeJ (C3H), and (D) ILS×ISS. The SNP distribution profiles were generated by plotting the number of SNPs in 250 kb bins. Vertical red lines mark the approximate positions of recombination (corresponds to figure 2). Region covered by *Qrr1p* (horizontal line), candidate genes in *Qrr1d* (yellow bars), and position of tRNA clusters (triangles) are shown above the graphs. The B6×D2, B6×BALB, and B6×C3H crosses have moderate to high SNP counts throughout *Qrr1*. In the ILSxISS cross, *Qrr1p* is relatively SNP-rich but *Qrr1d* is SNP-sparse.

SNPs were counted for all four pairs of parental haplotypes—*B* vs *D*, *B* vs *H*, *B* vs *C*, and *L* vs *S*—and SNP profiles for the four crosses were compared (figure 6). *Qrr1* is a highly polymorphic interval in the B6×D2 crosses. The flanking regions, however, have few SNPs (170–172.25 Mb proximally, and 177.5–179.5 Mb distally) and are almost identical-by-descent between B6 and D2. The B6×BALB crosses, despite being negative for the *trans*-effect, have moderate to high SNP counts in *Qrr1* and share a SNP profile somewhat similar to B6×D2 crosses. The B6×C3H crosses also have moderate to high SNP counts in *Qrr1*, with a relatively higher SNP count in *Qrr1d* compared to *Qrr1p*. In contrast, in the LXS, *Qrr1p* is more SNP-rich than *Qrr1d*. Most notably, the segments that harbor the tRNAs and candidates *Fmn2*, *Grem2*, and *Rgs7* are almost identical by descent between ILS and ISS. This SNP comparison indicates that the strongest *trans*-effect is from *Qrr1d*. A possible reason why the *trans*-effect is not detected in the CXB RI strains, despite being SNP rich in *Qrr1*, is that the crucial SNPs underlying the *trans*-QTLs may not be segregating in this cross or that undetected copy number variants make important contributions to the *Qrr1* effects. A final explanation may be that the small CXB dataset (13 strains) is simply underpowered.

### High-Ranking Candidates Based on Cross Specificity of *cis*-QTLs

We used the specificity of *cis*-QTLs in the multiple crosses to identify higher priority candidates in *Qrr1*. The assumption is that candidate genes whose transcripts have *cis*-QTLs (LOD score above 3) in the B6×D2 and B6×C3H crosses but not in the LXS and CXB RI strains are stronger candidates for *trans*-QTLs that are detected in the former two crosses but not in the latter two crosses. In contrast, *cis*-QTLs with the inverse cross specificity are less likely to underlie these *trans*-QTLs. Based on this criterion, there are four high-ranking candidates in *Qrr1p*—Purkinje cell protein 4-like 1 (*Pcp4l1*), prefoldin (*Pfdn2*), WD repeat domain 42 a (*Wdr42a*), and *Kcnj10* (table 3). There are only two high-ranking candidates in *Qrr1d*—formin 2 (*Fmn2*), an actin binding protein involved in cytoskeletal organization, and regulator of G-protein signaling 7 (*Rgs7*) (table 3).

Both *Fmn2* and *Rgs7* are almost exclusively expressed in the CNS and are high priority candidates for the CNS specific *trans*-QTLs. A point of distinction between the two candidates is that while expression of *Rgs7* maps as a *cis*-QTL only in the B6×D2 and B6×C3H crosses, expression of *Fmn2* maps as a *cis*-QTL in B6×D2 and B6×C3H crosses, and in the CXB RI strains in which the *trans*-effect is not detected (table 3). Based on the pattern of specificity of *cis*-QTLs in multiple crosses, *Rgs7* is a more appealing candidate. However, *Fmn2* has known missense SNPs that segregate in the B6×D2 (Glu610Asp, Pro1077Leu, Asp1431Glu) and B6×C3H crosses (Val372Ala). There are no known missense mutations in *Fmn2* in the CXB and LXS RI strains, and no known missense mutation in *Rgs7* in any of the four crosses.

### Partial Correlation Analysis

Linkage disequilibrium (LD) is a major confounding factor that limits fine-scale discrimination among physically linked candidates in a QTL. To further evaluate the two high-priority candidates in *Qrr1d*—*Fmn2* and *Rgs7*—we implemented a partial correlation analysis [68] in which the effect of genotype at *Qrr1d* was controlled. For this analysis, we computed the partial correlation coefficient between *cis*-regulated transcripts and each *trans*-regulated transcript after regression against the *Qrr1d* genotype. This partial correlation reveals residual variance that links *cis* candidates with *trans* targets, independent of genetic variance at *Qrr1d*. We computed the partial correlation between *Rgs7* and *Fmn2*, and 14 transcripts representative of the different GOs that map to *Qrr1d* (dataset S4). The highest partial correlations are between *Fmn2* and *Rnf6* (*r* = 0.68, *p*-value<10^−13^), *Atf4* (*r* = 0.6, *p*-value<10^−9^), *Asns* (*r* = 0.55, *p*-value<10^−7^), *Ube2d3* (*r* = 0.5, *p*-value<10^−6^), *Hnrpk* (*r* = 0.5, *p*-value = 10^−5^), *Rab2* (*r* = −0.5, *p*-value = 10^−5^), and *Gars* (*r* = 0.5, *p*-value = 10^−5^). The strongest correlate of *Fmn2* is *Rnf6*, a gene involved in regulating actin dynamics in axonal growth cones [69]. Although not unequivocal, this analysis provides stronger support for *Fmn2* than for *Rgs7*.

### Effect of *Fmn2* Deletion on Gene Expression

*Fmn2* is almost exclusively expressed in the nervous system [70] and is a strong candidate for a *trans*-effect specific to neural tissues. However, its precise function in the brain has not been established. *Fmn2*-null mice do not have notable CNS abnormalities [71], but to evaluate a possible role of *Fmn2* on expression of genes that map to *Qrr1d*, we generated array data from brains of *Fmn2*-null (*Fmn2^−/−^*) and coisogenic (*Fmn2^+/+^*) 129/SvEv controls. At a stringent statistical threshold (Bonferroni corrected *p*<0.05), only eight genes have significant expression differences between *Fmn2^−/−^* and *Fmn2^+/+^* genotypes (table 6). Five out of the eight genes, including *Pou6f1*, *Usp53*, and *Slc11a*, have *trans*-QTLs in *Qrr1d*. Deletion of *Fmn2* had the most drastic effect on the expression of the transcription factor gene *Pou6f1*, a gene implicated in CNS development and regulation of brain-specific gene expression [72],[73]. Expression of *Pou6f1* maps as a *trans*-QTL (at LOD score of 3) to *Qrr1d* in the hippocampus dataset, and its expression was down-regulated more than 44-fold in the *Fmn2^−/−^* line. While the expression analysis of *Fmn2*-null mice does not definitively link all the *trans*-QTLs to *Fmn2*, variation in this gene is likely to underlie some of the *trans*-QTLs in *Qrr1d*. The possible compensatory mechanism in the *Fmn2*-null CNS, and the different genetic background of the mice (129/SvEv) are factors that may have contributed to the weak detection of *trans*-effects in the knockout line.

**Table 6:** Genes that have significant expression difference between *Fmn2* ^+/+^ and *Fmn2* ^−/−^.

Gene	ProbeID^a^	Chr^b^	*Fmn2* ^+/+^ c	*Fmn2* ^−/−^ c	Fold^d^	p^e^	LOD^f^	Dataset^f^
*Pou6f1*	ILM6200168	15	11.96	6.48	45	3×10^−6^	3.0	BXD Hippocampus
*Zfp420*	ILM2570632	7	10.12	7.70	5	0.002		
*Txnl1*	ILM2850148	18	10.72	6.70	16	0.002	3.0	B6D2F2 striatum
*Usp53*	ILM103190068	3	7.17	9.32	4	0.009	3.3	BXD Hippocampus
*LOC331139*	ILM103170273	4	14.45	10.59	15	0.01		
*Slc11a2*	ILM104050242	15	9.92	9.17	2	0.02	3.9	BXD Hippocampus
*Pgbd5*	ILM103940435	8	13.40	12.12	2	0.02	3.3	BXD HBP Striatum
*6330569M22Rik*	ILM104570300	3	6.42	10.63	18	0.03		

a Illumina probe ID.

b Physical location of gene.

c Average expression signal in Fmn2-null and wild-type lines.

d Fold difference in expression between Fmn2-null and wild-type lines

e Bonferroni adjusted *p*-values; corrected for 46,620 tests.

f Highest LOD in *Qrr1* and dataset in which transcript has highest LOD in *Qrr1*.

### Sub-Cellular Localization of FMN2 Protein in Hippocampal Neurons

**Figure 7:**
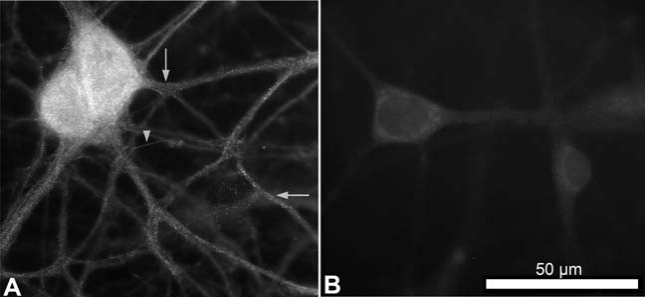
Expression of FMN2 protein in hippocampal neurons. (A) Neurons exhibited pronounced fine granular immunoreactivity for FMN2. The cell body had the strongest signal. The fine granular staining extended into apical and distal dendrites (arrows). Thin axon-like processes were also labeled (arrow head). (B) The fine granular staining is not detected in controls of sister cultures processed in parallel without the first antibody.

We examined the intracellular distribution of FMN2 protein in neurons using immunocytochemical techniques. All hippocampal pyramidal neurons on a culture dish exhibited distinct and fine granular immunoreactivity for FMN2. The cell body itself had the strongest signal (figure 7A). This fine punctate labeling extended into proximal dendrites and could be followed into distal dendrites. In some instances very thin processes, possibly the axons, were also labeled.

### Linking Expression and Classical QTLs: *Szs1*

**Figure 8:**
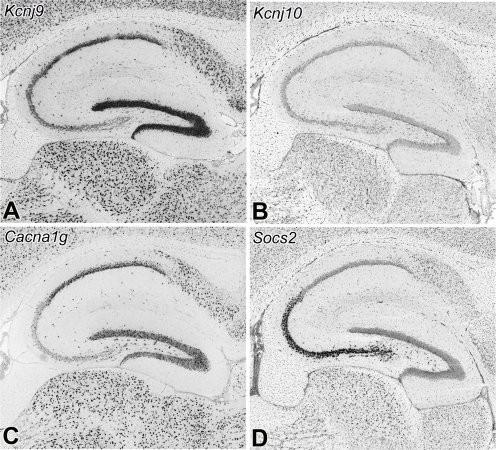
Expression patterns of seizure related genes with *cis*- and *trans*-QTLs in *Qrr1p*. Candidate gene *Kcnj9* (A) has heavy expression in neurons. *Kcnj9* shows a regionally restricted expression in the hippocampus with intense labeling in dentate gyrus, strong labeling in CA1, and relatively weak labeling in CA2 and CA3. Candidate gene *Kcnj10* (B) has a more diffused pattern and expressed primarily in glial cells. There is almost no labeling for *Kcnj10* in the hippocampus. Transcripts of seizure-related genes, *Cacna1g* (C) and *Socs2* (D), have trans-QTLs in *Qrr1p*. Both genes show high expression in neurons. *Cacna1g* matches the expression of *Kcnj9* with strong labeling in dentate gyrus and CA1, and weak labeling in CA2 and CA3. *Socs2* complements the expression of *Kcnj9* and *Cacna1g* with intense labeling in CA2 and CA3. *In Situ* expression data are from the Allen Brain Atlas.

The strong *trans*-effect that *Qrr1* has on gene expression is a likely basis for the classical QTLs that map to this region. For example, the major seizure susceptibility QTL (*Szs1*) has been precisely narrowed to *Qrr1p* [74]. We found that 10 genes already known to be associated with seizure or epilepsy have *trans*-QTLs with peak LOD scores near *Szs1* and in *Qrr1p*. These include *Scn1b*, *Cacna1g*, *Pnpo*, and *Dapk1* (Table S2) [75]–[84]. In every case, the *D* allele has the positive additive effect on the expression of these seizure related transcripts, increasing expression 5% to 20%. The two potassium channel genes, *Kcnj9* and *Kcnj10*, are the primary candidates [74]. Both are strongly *cis*-regulated. The tight linkage between these genes (within 100 kb) limits further genetic dissection, but *in situ* expression data from the Allen Brain Atlas (ABA, www.brain-map.org) provides us with a powerful complementary approach to evaluate these candidates [85]. *Kcnj9* (figure 8A) is expressed most heavily in neurons within the dentate gyrus, whereas *Kcnj10* (figure 8B) is expressed diffusely in glial cells in all parts of the CNS. The seizure-related transcripts with *trans*-QTLs near *Szs1* are most highly expressed in neurons, and all have comparatively high expression in the hippocampus. Furthermore, expression patterns of six of the seizure transcripts that map to *Qrr1p* show spatial correlations with *Kcnj9*. *Dapk1* and *Cacna1g* (figure 8C) have expression pattern that match *Kcnj9* with strong labeling in the dentate gyrus and CA1, and weaker labeling in CA2 and CA3. In contrast, *Socs2* (figure 8D), *Adora1*, *Pnpo*, and *Kcnma1* complement the expression of *Kcnj9* with comparatively strong expression in CA2 and CA3, and weak expression in CA1 and dentate gyrus.

## Discussion

*Qrr1* is a complex regulatory region that modulates expression of many genes and classical phenotypes. By exploiting a variety of microarray datasets and by applying a combination of high-resolution mapping, sequence analysis, and multiple cross analysis, we have dissected *Qrr1* into segments that are primarily responsible for variation in the expression of functionally coherent sets of transcripts. The distal portion of *Qrr1* (*Qrr1d*) has a strong *trans*-effect on RNA metabolism, translation, tRNA aminoacylation, and transcripts that are transported into neuronal dendrites. *Fmn2*, *Rgs7*, and a cluster of tRNAs are strong candidates in *Qrr1d*. We analyzed gene expression changes in the CNS of *Fmn2*-null mice and detected a profound effect on the expression of a small number of transcripts that map to *Qrr1d*, particularly on the expression of the transcription factor *Pou6f1*. We have shown that the FMN2 protein is highly expressed in the cell body and processes of neurons, and is a high priority candidate in *Qrr1d*.

### *Kcnj9* vs. *Kcnj10* and Seizure Susceptibility

The two inwardly rectifying potassium channel genes—*Kcnj9* and *Kcnj10*—are strong candidates for the seizure susceptibility QTL in *Qrr1p* that has been unambiguously narrowed to the short interval from *Atp1a2* to *Kcnj10* [74]. In BXD CNS datasets, *Qrr1* also modulates the expression of a set of genes implicated in the etiology of seizure and epilepsy, including *Pnpo*, *Scn1b*, *Kcnma1*, *Socs2*, and *Cacna1g*. Polymorphisms in the *Kcnj9/Kcnj10* interval that influence expression of these genes are excellent candidates for the *Szs1* locus.

The *in situ* expression data in the ABA shows a striking spatial correlation between expression of *Kcnj9* and other seizure-related transcripts that have *trans*-QTLs in *Qrr1p*. The complementary expression of *Kcnj9* and the seizure-related transcripts (figure 8) make *Kcnj9* a stronger candidate than *Kcnj10*. *Kcnj9* has over a 2-fold higher expression in D2 [our data],[and cf. 26,86], a seizure prone strain, compared to B6, a relatively seizure resistant strain, suggesting that the proximal cause of *Szs1* may be high expression of this gene, perhaps due to the promoter polymorphism discovered by Hitzemann and colleagues [26].

### Multiple Loci in a Major QTL Interval

Fine mapping of complex traits have often yielded multiple constituent loci within a QTL interval [87],[88]. Our mapping analyses of expression traits also show that multiple gene variants, rather than one master regulatory gene, cause the aggregation of expression QTLs in *Qrr1*. Subgroups of genes with tight coexpression can be dissected from the dense cluster of QTLs. Most notable is the strong *trans*-regulatory effect of *Qrr1d* on genes involved in amino acid metabolism and translation, including a host of ARS transcripts. However, there are limits to our ability to dissect *Qrr1*, and genes associated with protein degradation and RNA metabolism map throughout the region. In part this may be due to inadequate mapping resolution, but it may also reflect clusters of functionally related loci and genes [89]. At this stage we are also unable to discern whether there is a single or multiple QTLs within *Qrr1d*. While it is likely that a single QTL modulates the expression of the ARS, there may be additional gene variants in *Qrr1d* that modulate other transcripts involved in translation and RNA metabolism. With increased resolving power it may be possible to further subdivide transcripts that map to *Qrr1p* and *Qrr1d* into smaller functional modules.

There may be multiple loci in *Qrr1* that modulate different stages of protein metabolism in the CNS. Maintenance of cellular protein homeostasis requires finely tuned cross talk between transcription and RNA processing, the translation machinery, and protein degradation [90]–[92], gene functions highly overrepresented among the transcripts that map to *Qrr1*. While these are generic cellular processes, there are unique demands on protein metabolism in the nervous system. Neurons are highly polarized cells and specialized mechanisms are in place to manage local protein synthesis and degradation in dendrites and axons [93]. The nervous system is also particularly sensitive to imbalances in protein homeostasis [94],[95], a possible reason why the *trans*-effects of *Qrr1* are detected only in neural tissues.

### Candidates in *Qrr1d* and Possible Links with Local Protein Synthesis

Transfer RNAs are direct biological partners of the ARS, and the cluster of tRNAs in the highly polymorphic intergenic region of *Qrr1d* (figure 6) is an enticing candidate. In addition to their role in shuttling amino acids, tRNAs also act as sensors of cellular amino acid levels and regulate transcription of genes involved in amino acid metabolism and the ARS [66]. There is tissue specificity in the expression of different tRNA isoforms [96], and we speculate that the tRNA cluster in *Qrr1d* is specifically functional in neural tissues.

*Rgs7*, a member of the RGS (regulator of G-protein signaling) family, is another high-ranking candidate in *Qrr1d*. RGS proteins are important regulators of G-protein mediated signal transduction. *Rgs7* is predominantly expressed in the brain and has been implicated in regulation of neuronal excitability and synaptic transmission [97],[98]. Although RGS proteins are usually localized in the plasma membrane, RGS7 has been found to shuttle between the membrane and the nucleus [99]. This implies a role for RGS7 in gene expression regulation in response to external stimuli.

Our final high-ranking candidate in *Qrr1d* is *Fmn2*. It codes for an actin binding protein exclusively expressed in the CNS and oocytes, and is involved in the establishment of cell polarity [70],[71]. In *Drosophila*, the formin homolog, cappuccino, has a role in RNA transport and in localizing the staufen protein to oocyte poles [100]–[102]. It is possible that FMN2 has parallel functions in mammalian neurons. Interestingly, Staufen 2 (*Stau2*), a gene involved in RNA transport to dendrites [62], maps to *Qrr1d* in BXD CNS datasets. Furthermore, deletion of formin homologs in yeast results in inhibition of protein translation [103], compelling evidence for an interaction between the protein translation system and formins. Evidence for a role for *Fmn2* in dendrites also comes from our immunocytochemical analysis that clearly demonstrates the expression of FMN2 protein in dendrites. Taken together, *Fmn2* is a functionally relevant candidate gene in *Qrr1d* and may be related to RNA transport and protein synthesis in the CNS.

## Methods

### Microarray Datasets

The microarray datasets used in this study (table 2) were generated by collaborative efforts [46], [47], [49]–[52]. All datasets can be accessed from www.genenetwork.org. They provide estimates of global mRNA abundance in neural and non-neural tissues in the BXD, LXS, and CXB RI strains, B6D2F2 intercrosses, and B6C3HF2 intercrosses. Detailed description of each set, tissue acquisition, RNA extraction and array hybridization methods, and data processing and normalization methods are provided in the “Info” page linked to each dataset. In brief, the datasets are:

1. BXD CNS transcriptomes: The BXD CNS datasets measure gene expression in the forebrain and midbrain (INIA Forebrain), striatum (HBP/Rosen Striatum and HQF Striatum), hippocampus (Hippocampus Consortium and UMUTAffy Hippocampus), cerebellum (SJUT Cerebellum mRNA), and eye (Hamilton Eye) of BXD RI strains (table 2). The INIA Brain and HBP/Rosen Striatum datasets have been described in Peirce et al. [47]. The Hippocampus Consortium dataset measures gene expression in the adult hippocampus of 69 BXD RI strains, the parental B6 and D2 strains, and F1 hybrids. The SJUT Cerebellum dataset measures gene expression in the adult cerebellum of 45 BXD RI strains, parental strains, and F1 hybrids. The Eye dataset measures gene expression in the eyes of 64 BXD RI strains, parental strains, and F1 hybrids. The HQF BXD Striatum is one of the newest datasets and was generated on Illumina Sentrix Mouse–6.1 arrays. It is similar to the HBP/Rosen Striatum and measures gene expression in the striatum of 54 BXD RI strains, parental strains, and F1 hybrids.
2. BXD non-neural transcriptomes: The non-neural BXD array sets measure gene expression in the liver (UNC Liver) of 40 BXD strains, kidney (Kidney Consortium) of 53 BXD strains, and hematopoietic stem cells (GNF Hematopoietic Cells) of 30 BXD strains [49],[50].
3. LXS hippocampus transcriptome: The LXS Hippocampus dataset measures gene expression in the adult hippocampus of 75 LXS RI strains and the parental ILS and ISS strains.
4. B6D2F2 CNS transcriptomes: The B6D2F2 datasets measure gene expression in the whole brain (OHSU/VA Brain), and striatum (OHSU/VA Striatum) of B6×D2 F2 intercrosses [47],[52]. The whole brain dataset comprises of samples from 56 F2 animals, and the striatum dataset comprises of samples from 58 F2 animals.
5. B6C3HF2 transcriptomes: These datasets were generated from large numbers of B6×C3H F2 intercross progeny and assayed using Agilent arrays [51]. These datasets have been described in Yang et al [51].

### Mouse Strains and Genotype Data

The conventional BXD RI strains were derived from the B6 and D2 inbred mice [104],[105]. The newer sets of advanced RI strains were derived by inbreeding intercrosses of the RI strains [57]. The parental B6 and D2 strains differ significantly in sequence and have approximately 2 million informative SNP. A subset of 14,000 SNPs and microsatellite markers have been used to genotype the BXD strains [106],[107]. We used 3,795 informative markers for QTL mapping. Thirty such informative markers are in *Qrr1* and we queried these markers to identify strains with recombinations in *Qrr1*; genes with strong *cis*-QTLs (*Sdhc*, *Atp1a2*, *Dfy*, and *Fmn2*) were used as additional markers. Smaller sub-sets of markers were used to genotype the two F2 panels (total of 306 markers for the whole brain, and 75 markers for the striatum F2 datasets).

The LXS RI strains were derived from the ILS and ISS inbred strains. They have been genotyped using 13,377 SNPs, and some microsatellite markers [108]. 2,659 informative SNPs and microsatellite markers were used for QTL mapping.

The CXB panel consists of 13 RI strains derived from C57BL/6By and BALB/cBy inbred strains. A total of 1384 informative markers were used for QTL mapping.

The B6×C3H/HeJ F2 intercrosses have been genotyped using 13,377 SNPs and microsatellite markers, and 8,311 informative markers were used for QTL mapping.

### Animals and Tissue Acquisition

Majority of the BXD and LXS tissues (cerebellum, eye, forebrain, hippocampus, kidney, liver, and striatum for the HQF Illumina dataset) were dissected at the University of Tennessee Health Science Center (UTHSC). Mice were housed at the UTHSC in pathogen-free colonies, at an average of three mice per cage. All animal procedures were approved by the Animal Care and Use Committee. Mice were killed by cervical dislocation, and tissues were rapidly dissected and placed in RNAlater (Ambion, www.ambion.com) and kept overnight at 4° C, and subsequently stored at −80 degree C. Tissue were then processed at UTHSC or shipped to other locations for processing.

### RNA Isolation and Sample Preparation

For the tissues that were processed at UTHSC (all BXD and LXS CNS tissues except HBP Affymetrix striatum), RNA was isolated using RNA STAT-60 (Tel-Test Inc., www.tel-test.com) as per manufacturer's instructions. Samples were then purified using standard sodium acetate methods prior to microarray hybridization. The eye samples required additional purification steps to remove eye pigment; this was done using the RNeasy MinElute Cleanup Kit (Qiagen, www.qiagen.com). RNA purity and concentration was evaluated with a spectrophotometer using 260/280 nm absorbance ratio, and RNA quality was checked using Agilent Bioanalyzer 2100 prior to hybridization. Array hybridizations were then done according to standard protocols.

### Microarray Probe Set Annotation

We have re-annotated a majority of Affymetrix probe sets to ensure more accurate description of probe targets. Each probe set represents a concatenations of eleven 25-mer probes, and these have been aligned to the NCBI built 36 version of the mouse genome (mm8 in UCSC Genome Browser) by BLAT analysis. We have also re-annotated the Illumina probes and incorporated these annotations into GeneNetwork. Each probe in the Illumina Mouse–6 and Mouse–6.1 arrays is 50 nucleotides in length, and these have been aligned to NCBI built 36.

### QTL Mapping

We used the strain average expression signal detected by a probe or probe set. QTL mapping was done for all transcripts using QTL Reaper [47]. The mapping algorithm combines simple regression mapping, linear interpolation, and standard Haley-Knott interval mapping [109]. QTL Reaper performs up to a million permutations of an expression trait to calculate the genome-wide empirical *p*-value and the LOD score associated with a marker. We selected only those transcripts that have highest LOD scores, i.e., genome-wide adjusted best *p*-values, on markers located on Chr 1 from 172 to 178 Mb. This selected transcripts that are primarily modulated by *Qrr1* but excluded transcripts that have QTLs in *Qrr1* but have higher LOD scores on markers located on other chromosomal regions. *Cis*- and *trans*-QTLs were distinguished based on criteria described by Peirce et al. [47]. To identify *trans*-QTLs common to multiple datasets, we selected probes/probe sets that target the same genes and have peak LOD scores within 10 Mb in the different datasets.

### Screening Local QTLs

We screened all Affymetrix probe sets with *cis*-QTLs in *Qrr1* for SNPs in target sequences. This step was taken to identity false *cis*-QTLs caused by differences in hybridization. As probe design is based on the B6 sequence, such spurious *cis*-QTLs show high expression for the *B* allele, and low expression for the *D* allele. Our screening identified only two probe sets in which SNPs result in spurious local QTLs—1429382_at (*Tomm40l*), and 1452308_a_at (*Atp1a2*). The majority of *cis*-QTLs in *Qrr1* are likely to be due to actual differences in mRNA abundance. We did not detect a bias in favor of the *B* allele on *cis*-regulated expression and the ratio of transcripts with *B*- and *D*- positive additive effects is close to 1∶1.

### Analysis of Allele-Specific Expression Difference

To measure expression difference between the *B* and *D* alleles, we exploited transcribed SNPs to capture allelic expression difference in F1 hybrids [56] using a combination of RT-PCR and a single base extension technology (SNaPshot, Applied Biosystems, www.appliedbiosystems.com). For each transcript we analyzed, Primer 3 [110] was used to design a pair of PCR primers that target sequences on the same exon and flanking an informative SNP.

We prepared four pools of RNA from the hippocampus, and four pools of genomic DNA from the spleen of F1 hybrids (male and female B6×D2 and D2×B6 F1 hybrids). To avoid contamination by genomic DNA, the four RNA pools were treated with Turbo DNase (Ambion, www.ambion.com), and then first strand cDNA was synthesized (GE Healthcare, www.gehealthcare.com). The genomic DNA samples were used as controls, and both cDNA and genomic DNA samples were tested concurrently using the same assay to compare expression levels of *B* and *D* transcripts.

We amplified the cDNA and genomic DNA samples using GoTaq Flexi DNA polymerase (Promega Corporation, www.promega.com). PCR products were purified using ExoSap-IT (USB Corporation, www.usbweb.com) followed by SNaPshot to extend primer by a single fluorescently labeled ddNTPs. Fluorescently labeled products were purified using calf intestinal phosphatase (CIP, New England BioLabs, www.neb.com) and separated by capillary electrophoresis on ABI3130 (Applied Biosystems). Quantification was done using GeneMapper v4.0 software (Applied Biosystems), and transcript abundance was measured by peak intensities associated with each allele. Ratio of *B* and *D* allele in both cDNA and gDNA pools was computed, and t-test (one tail, unequal variance) was done to validate expression difference and polarity of parental alleles.

### SNP Analysis in Multiple Crosses

GeneNetwork has compiled SNP data from different sources—Celera (http://www.celera.com), Perlegen/NIEHS (http://mouse.perlegen.com/mouse/download.html), BROAD institute (http://www.broad.mit.edu/snp/mouse), Wellcome–CTC [107], dbSNP, and Mouse Phenome Database (http://www.jax.org/phenome/SNP). SNP counts were done on the GeneNetwork SNP browser.

### Partial Correlation Analysis

A partial correlation is the correlation between *X* and *Y* conditioned on one or more control variables. In this study, first order partial correlation was used to detect the interaction between trans-regulated transcripts and *cis*-regulated candidate genes conditioned on the genotype (marker rs8242481 at 175.058 Mb). If x, y and z are *trans*-regulated transcripts, *cis*-regulated transcript, and genotype in the QTL, respectively, then the first order partial correlation coefficient is calculated as—
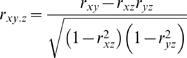
where *r_xy_* can be either Pearson correlation or Spearman's rank correlation between x and y. We employed the Spearman's rank correlation because the expression levels of many transcripts do not follow a normal distribution.

The significance of a partial correlation with *n* data points was assessed with a two-tailed *t* test on 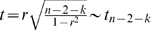 where *r* is the first order correlation coefficient, and *k* is the number of variables on which we are conditioning.

### Immunocytochemistry

Cultured hippocampal neurons from male B6 mice, prepared as described in Schikorski et al. [111] and cultured for 23 days, were fixed with 4% paraformaldehyde and 0.1% glutaraldehyde in HEPES buffered saline (pH 7.2) for 15 min. Cell membranes were permeabilized with 0.1% triton X-100 and unspecific binding sites were quenched with 10% BSA for 20 min at room temperature (RT). Neurons were incubated with a polyclonal anti-FMN2 antibody (Protein Tech Group, www.ptglab.com) diluted to 0.3 µg/ml at RT overnight. An anti-rabbit antibody raised in donkey (1∶500, Invitrogen; http://www.invitrogen.com) conjugated with the fluorescent dye Alexa488 was used for the detection of the first antibody. All regions of interest were photographed with identical illumination and camera settings to allow for a direct comparison of the staining in labeled and control neurons.

### Fmn2^−/−^ and Fmn2^+/+^ Microarray Analysis

The *Fmn2^−/−^* mice were generated using 129/SvEv (now strain 129S6/SvEvTac) derived TC-1 embryonic stem cells. Chimeric mice were backcrossed to 129/SvEv [70]. The *Fmn2*-null and littermate controls are therefore coisogenic. To validate the isogenicity of regions surrounding the targeted locus [112], we genotyped the *Fmn2^+/+^*, *Fmn2^+/−^*, and *Fmn2^−/−^* mice using ten microsatellite markers located on, and flanking *Fmn2* (markers distributed from 172 Mb to 182 Mb). These markers are *D1Mit455*, *D1Mit113*, *D1Mit456*, *D1Mit356*, *D1Mit206*, *D1Mit355*, *D1Mit150*, *D1Mit403*, *D1Mit315*, and *D1Mit426*. With the exception of a marker at *Fmn2* (*D1Mit150*), all alleles in null, heterozygote, and wildtype animals were identical.

RNA was isolated from whole brain samples of *Fmn2^+/+^* and *Fmn2^−/−^* mice, and assayed on Illumina Mouse-6 array slides (six samples per slide). We compared five samples from *Fmn2^−/−^* nulls, and five samples from *Fmn2^+/+^* wildtype. Equal numbers of each genotypes were placed on each slide to avoid batch confounds. Microarray data were processed using both raw and rank invariant protocols provided by Illumina as part of the BeadStation software suite (www.illumina.com). We subsequently log-transformed expression values and stabilized the variance of each array. To identify genes with significant expression difference between the *Fmn2^−/−^* and *Fmn2^+/+^* cases, we carried out two-tailed *t*-tests and applied a Bonferroni correction for multiple testing, and selected probes with a minimum adjusted *p*-value<0.05.

### Bioinformatics Tools

Classical QTLs counts are based on the April 2008 version of Mouse Genome Informatics (MGI: www.informatics.jax.org) [113]. Search for tRNAs was done using tRNAscan-SE 1.21 (http://lowelab.ucsc.edu/tRNAscan-SE/) [65]. GO analysis was done using the analytical tool DAVID 2007 (http://david.abcc.ncifcrf.gov/) [114]. Overrepresented GO terms were identified and statistical significance of enrichment was calculated using a modified Fisher's Exact Test or EASE score [115]. We used the Allen Brain Atlas to analyze expression pattern in the brain of young C57BL/6J male mice (www.brain-map.org) [85],[116].

### Control for Non-Syntenic Association and Paralogous Region

In RI strains, non-syntenic associations can lead to LD between distant loci [89],[106]. In the BXDs, we detected such non-syntenic associations between markers in *Qrr1* and markers on distal Chr 2 and proximal Chr 15. As a result of these associations, some transcripts that have strong *cis*- or *trans*-QTLs in *Qrr1* tend to have weak LOD peaks, usually below the suggestive threshold, on distal Chr 2 and proximal Ch15. However, there is no bias for genes located in these intervals in LD with *Qrr1* to have *trans*-QTLs in *Qrr1*.

The *Qrr1* segment has been reported to have paralogues on mouse Chrs 1 (proximal region), 2, 3, 6, 7, 9, and 17 [117],[118]. We examined if the *trans*-QTLs in *Qrr1* are of genes located in these paralogous regions. However, genes located in the paralogous regions are not overrepresented among the *trans*-QTL.

## Supporting Information

Table S1 Number of classical QTLs in Qrr1 and in hundred other chromosomal intervals.
(0.23 MB DOC)

Table S2 Transcripts of genes associated with seizure or epilepsy that have *trans*-QTLs in *Qrr1p* near the seizure susceptibility QTL.
(0.05 MB DOC)

Dataset S1 Precision of Cis-QTLs in Qrr1.
(0.13 MB XLS)

Dataset S2 Gene ontology analysis of transcripts that map to *Qrr1p* and *Qrr1d* in the BXD hippocampus dataset.
(0.03 MB XLS)

Dataset S3 tRNAs in *Qrr1d*.
(0.09 MB XLS)

Dataset S4 Partial correlation analysis.
(0.04 MB XLS)
