# Supplementary material for: Dissection of a QTL Hotspot on Mouse Distal Chromosome 1 that Modulates Neurobehavioral Phenotypes and Gene Expression
Source: PLoS Genet. 2008 Nov 14;4(11):e1000260. doi: 10.1371/journal.pgen.1000260 (PMC2577893; doi:10.1371/journal.pgen.1000260)
Supplement: Table S1 — Number of classical QTLs in Qrr1 and in hundred other chromosomal intervals. (0.23 MB DOC) [file pgen.1000260.s001.doc]

**Table S1: Number of classical QTLs in *Qrr1* and in hundred other chromosomal intervals**

| **Chr** | **Start**  **(Mb)** | **End**  **(Mb)** | **Length**  **(Mb)** | **Genes** | **QTLs** | **QTL/Mb** | **QTL/gene** |
| --- | --- | --- | --- | --- | --- | --- | --- |
| 1 | 5 | 25 | 20 | 160 | 8 | 0.4 | 0.05 |
| 1 | 30 | 46 | 16 | 164 | 2 | 0.1 | 0.01 |
| 1 | 51 | 66 | 15 | 170 | 10 | 0.7 | 0.06 |
| 1 | 70 | 85 | 15 | 169 | 16 | 1.1 | 0.09 |
| 1 | 92 | 114 | 22 | 155 | 14 | 0.6 | 0.09 |
| 1 | 125 | 139 | 14 | 170 | 10 | 0.7 | 0.06 |
| 1 | 172 | 178 | 6 | 164 | 32 | 5.3 | 0.20 |
| 1 | 180 | 197 | 17 | 169 | 15 | 0.9 | 0.09 |
| 2 | 15 | 26 | 11 | 159 | 6 | 0.5 | 0.04 |
| 2 | 30 | 37 | 7 | 162 | 9 | 1.3 | 0.06 |
| 2 | 50 | 72 | 22 | 165 | 22 | 1.0 | 0.13 |
| 2 | 83 | 87 | 4 | 169 | 6 | 1.5 | 0.04 |
| 2 | 95 | 113 | 18 | 167 | 8 | 0.4 | 0.05 |
| 2 | 119 | 128 | 9 | 163 | 6 | 0.7 | 0.04 |
| 2 | 138 | 152.5 | 14.5 | 162 | 13 | 0.9 | 0.08 |
| 2 | 164 | 175 | 11 | 170 | 12 | 1.1 | 0.07 |
| 3 | 15 | 38 | 23 | 170 | 5 | 0.2 | 0.03 |
| 3 | 50 | 68 | 18 | 168 | 9 | 0.5 | 0.05 |
| 3 | 75 | 89 | 14 | 164 | 7 | 0.5 | 0.04 |
| 3 | 90 | 95 | 5 | 172 | 1 | 0.2 | 0.01 |
| 3 | 98 | 108 | 10 | 160 | 11 | 1.1 | 0.07 |
| 3 | 120 | 138 | 18 | 164 | 10 | 0.6 | 0.06 |
| 4 | 20 | 42 | 22 | 166 | 15 | 0.7 | 0.09 |
| 4 | 55 | 73 | 18 | 153 | 16 | 0.9 | 0.10 |
| 4 | 95 | 110 | 15 | 160 | 20 | 1.3 | 0.13 |
| 4 | 115 | 121 | 6 | 161 | 6 | 1.0 | 0.04 |
| 4 | 130 | 137 | 7 | 165 | 12 | 1.7 | 0.07 |
| 5 | 21 | 32 | 11 | 163 | 5 | 0.5 | 0.03 |
| 5 | 35 | 62 | 27 | 158 | 22 | 0.8 | 0.14 |
| 5 | 70 | 89 | 19 | 160 | 5 | 0.3 | 0.03 |
| 5 | 94 | 106 | 12 | 161 | 20 | 1.7 | 0.12 |
| 5 | 112 | 122 | 10 | 158 | 10 | 1.0 | 0.06 |
| 5 | 130 | 139 | 9 | 179 | 5 | 0.6 | 0.03 |
| 6 | 30 | 42 | 12 | 159 | 3 | 0.3 | 0.02 |
| 6 | 45 | 54 | 9 | 161 | 7 | 0.8 | 0.04 |
| 6 | 65 | 70 | 5 | 184 | 2 | 0.4 | 0.01 |
| 6 | 85 | 93 | 8 | 167 | 13 | 1.6 | 0.08 |
| 6 | 100 | 120 | 20 | 159 | 13 | 0.7 | 0.08 |
| 6 | 130 | 142 | 12 | 166 | 8 | 0.7 | 0.05 |
| 7 | 4 | 10 | 6 | 180 | 4 | 0.7 | 0.02 |
| 7 | 20 | 25 | 5 | 183 | 0 | 0.0 | 0.00 |
| 7 | 29 | 33 | 4 | 182 | 5 | 1.3 | 0.03 |
| 7 | 51 | 54 | 3 | 164 | 4 | 1.3 | 0.02 |
| 7 | 75 | 92 | 17 | 152 | 10 | 0.6 | 0.07 |
| 7 | 100 | 110 | 10 | 164 | 7 | 0.7 | 0.04 |
| 7 | 130 | 139 | 9 | 180 | 17 | 1.9 | 0.09 |
| 8 | 12 | 24 | 12 | 168 | 1 | 0.1 | 0.01 |
| 8 | 30 | 49 | 19 | 169 | 10 | 0.5 | 0.06 |
| 8 | 66 | 75 | 9 | 178 | 7 | 0.8 | 0.04 |
| 8 | 80 | 91 | 11 | 165 | 7 | 0.6 | 0.04 |
| 8 | 105 | 113 | 8 | 159 | 2 | 0.3 | 0.01 |
| 8 | 116 | 130 | 14 | 163 | 7 | 0.5 | 0.04 |
| 9 | 12 | 22 | 10 | 188 | 1 | 0.1 | 0.01 |
| 9 | 36 | 40 | 4 | 169 | 8 | 2.0 | 0.05 |
| 9 | 43 | 54 | 11 | 169 | 17 | 1.5 | 0.10 |
| 9 | 63 | 76 | 13 | 158 | 11 | 0.8 | 0.07 |
| 9 | 82 | 100 | 18 | 165 | 17 | 0.9 | 0.10 |
| 9 | 108 | 118 | 10 | 163 | 5 | 0.5 | 0.03 |
| 10 | 10 | 30 | 20 | 176 | 10 | 0.5 | 0.06 |
| 10 | 33 | 53 | 20 | 153 | 5 | 0.3 | 0.03 |
| 10 | 60 | 77 | 17 | 170 | 16 | 0.9 | 0.09 |
| 10 | 85 | 100 | 15 | 154 | 8 | 0.5 | 0.05 |
| 10 | 111 | 127 | 16 | 163 | 21 | 1.3 | 0.13 |
| 11 | 15 | 34 | 19 | 170 | 14 | 0.7 | 0.08 |
| 11 | 43 | 52 | 9 | 165 | 5 | 0.6 | 0.03 |
| 11 | 60 | 69 | 9 | 164 | 15 | 1.7 | 0.09 |
| 11 | 78 | 87 | 9 | 162 | 14 | 1.6 | 0.09 |
| 11 | 96 | 100 | 4 | 171 | 7 | 1.8 | 0.04 |
| 11 | 110 | 119 | 9 | 160 | 11 | 1.2 | 0.07 |
| 12 | 8 | 25 | 17 | 163 | 4 | 0.2 | 0.02 |
| 12 | 31 | 57 | 26 | 168 | 11 | 0.4 | 0.07 |
| 12 | 70 | 83 | 13 | 167 | 11 | 0.8 | 0.07 |
| 12 | 90 | 109 | 19 | 168 | 12 | 0.6 | 0.07 |
| 12 | 115 | 117 | 2 | 157 | 1 | 0.5 | 0.01 |
| 13 | 10 | 22 | 12 | 175 | 7 | 0.6 | 0.04 |
| 13 | 32 | 49 | 17 | 154 | 11 | 0.6 | 0.07 |
| 13 | 55 | 65.5 | 10.5 | 174 | 8 | 0.8 | 0.05 |
| 13 | 72 | 95 | 23 | 169 | 13 | 0.6 | 0.08 |
| 13 | 100 | 119 | 19 | 169 | 11 | 0.6 | 0.07 |
| 14 | 14 | 31 | 17 | 167 | 2 | 0.1 | 0.01 |
| 14 | 43 | 50 | 7 | 160 | 1 | 0.1 | 0.01 |
| 14 | 60 | 71 | 11 | 163 | 4 | 0.4 | 0.02 |
| 14 | 76 | 109 | 33 | 167 | 7 | 0.2 | 0.04 |
| 15 | 9 | 38 | 29 | 164 | 6 | 0.2 | 0.04 |
| 15 | 47 | 73 | 26 | 155 | 23 | 0.9 | 0.15 |
| 15 | 82 | 94 | 12 | 161 | 12 | 1.0 | 0.07 |
| 16 | 8 | 19 | 11 | 171 | 3 | 0.3 | 0.02 |
| 16 | 25 | 39 | 14 | 171 | 7 | 0.5 | 0.04 |
| 16 | 46 | 65 | 19 | 153 | 4 | 0.2 | 0.03 |
| 16 | 72 | 91.5 | 19.5 | 160 | 9 | 0.5 | 0.06 |
| 17 | 5 | 17 | 12 | 166 | 9 | 0.8 | 0.05 |
| 17 | 25 | 31 | 6 | 169 | 11 | 1.8 | 0.07 |
| 17 | 39 | 50 | 11 | 151 | 17 | 1.5 | 0.11 |
| 17 | 65 | 84 | 19 | 162 | 5 | 0.3 | 0.03 |
| 18 | 5 | 25 | 20 | 165 | 3 | 0.2 | 0.02 |
| 18 | 33 | 39 | 6 | 170 | 7 | 1.2 | 0.04 |
| 18 | 48 | 67 | 19 | 162 | 14 | 0.7 | 0.09 |
| 19 | 3 | 7 | 4 | 173 | 2 | 0.5 | 0.01 |
| 19 | 12 | 22 | 10 | 165 | 7 | 0.7 | 0.04 |
| 19 | 28 | 41 | 13 | 168 | 9 | 0.7 | 0.05 |
| 19 | 45 | 60 | 15 | 155 | 17 | 1.1 | 0.11 |
