# Supplementary material for: Dissection of a QTL Hotspot on Mouse Distal Chromosome 1 that Modulates Neurobehavioral Phenotypes and Gene Expression
Source: PLoS Genet. 2008 Nov 14;4(11):e1000260. doi: 10.1371/journal.pgen.1000260 (PMC2577893; doi:10.1371/journal.pgen.1000260)
Supplement: Table S2 — Transcripts of genes associated with seizure or epilepsy that have trans-QTLs in Qrr1p near the seizure susceptibility QTL. (0.05 MB DOC) [file pgen.1000260.s002.doc]

**Table S2**: Transcripts of genes associated with seizure or epilepsy that have *trans*-QTLs in *Qrr1p* near the seizure susceptibility QTL

|  |  |  |  |  |  |  |
| --- | --- | --- | --- | --- | --- | --- |
| **Gene** | **ProbeID^a^** | **Chr^b^** | **Data set^c^** | **LRS^d^** | **B/D^e^** | **Reference^d^** |
|  |  |  |  |  |  |  |
| *Scn1b* | 1418738_at_A | Chr 7 | Forebrain | 3.5 | D | 75 |
| *Akt1* | 1416657_at_A | Chr 12 | Forebrain | 3.0 | D | 76 |
| *Cacna1g* | 1423365_at_A | Chr 11 | Forebrain | 2.8 | D | 77 |
| *Pnpo* | 1415793_at_A | Chr 11 | Cerebellum | 29 | D | 78 |
| *Dapk1* | 1426915_at_A | Chr 13 | Cerebellum | 6.3 | D | 79 |
| *Kcnma1* | 1457018_at_B | Chr 14 | Cerebellum | 3.7 | D | 80 |
| *Socs2* | 1449109_at_A | Chr 10 | Cerebellum | 3.7 | D | 81 |
| *Nsf* | 1422456_at_A | Chr 11 | Cerebellum | 3.3 | D | 82 |
| *Grm2* | 1435607_at_B | Chr 9 | Cerebellum | 3.3 | D | 83 |
| *Adora1* | 1435495_at | Chr 1 | Hippocampus | 3.0 | D | 84 |

**^a^**Affymetrix probe set ID **^b^**Physical location of gene; *Adora1* is located on Chr 1 at 136 Mb **^c^** Dataset in which transcript has highest *trans*-QTL in *Qrr1p*  **^d^**Highest LOD scores in *Qrr1p* **^e^**Allele that increases expression **^d^**Provided in the references section of main text
